# The Impact of the Culture Regime on the Metabolome and Anti-Phytopathogenic Activity of Marine Fungal Co-Cultures

**DOI:** 10.3390/md22020066

**Published:** 2024-01-27

**Authors:** Mohammed Zawad Reza, Ernest Oppong-Danquah, Deniz Tasdemir

**Affiliations:** 1 GEOMAR Centre for Marine Biotechnology (GEOMAR-Biotech), Research Unit Marine Natural Product Chemistry, GEOMAR Helmholtz Centre for Ocean Research Kiel, Wischhofstrasse 1-3, 24148 Kiel, Germany; zawadreza@gmail.com (M.Z.R.); eoppong-danquah@geomar.de (E.O.-D.); 2 Faculty of Mathematics and Natural Sciences, Kiel University, Christian-Albrechts-Platz 4, 24118 Kiel, Germany

**Keywords:** marine fungus, *Plenodomus influorescens*, *Pyrenochaeta nobilis*, co-cultivation, OSMAC, phytopathogen, metabolomics, Feature-Based Molecular Network

## Abstract

Co-cultivation, coupled with the OSMAC approach, is considered an efficient method for expanding microbial chemical diversity through the activation of cryptic biosynthetic gene clusters (BGCs). As part of our project aiming to discover new fungal metabolites for crop protection, we previously reported five polyketides, the macrolides dendrodolides E (**1**) and N (**2**), the azaphilones spiciferinone (**3**) and 8*α*-hydroxy-spiciferinone (**4**), and the *bis*-naphtho-*γ*-pyrone cephalochromin (**5**) from the solid Potato Dextrose Agar (PDA) co-culture of two marine sediment-derived fungi, *Plenodomus influorescens* and *Pyrenochaeta nobilis*. However, some of the purified metabolites could not be tested due to their minute quantities. Here we cultivated these fungi (both axenic and co-cultures) in liquid regime using three different media, Potato Dextrose Broth (PDB), Sabouraud Dextrose Broth (SDB), and Czapek-Dox Broth (CDB), with or without shaking. The aim was to determine the most ideal co-cultivation conditions to enhance the titers of the previously isolated compounds and to produce extracts with stronger anti-phytopathogenic activity as a basis for future upscaled fermentation. Comparative metabolomics by UPLC-MS/MS-based molecular networking and manual dereplication was employed for chemical profiling and compound annotations. Liquid co-cultivation in PDB under shaking led to the strongest activity against the phytopathogen *Phytophthora infestans*. Except for compound **1**, all target compounds were detected in the co-culture in PDB. Compounds **2** and **5** were produced in lower titers, whereas the azaphilones (**3** and **4**) were overexpressed in PDB compared to PDA. Notably, liquid PDB co-cultures contained meroterpenoids and depside clusters that were absent in the solid PDA co-cultures. This study demonstrates the importance of culture regime in BGC regulation and chemical diversity of fungal strains in co-culture studies.

## 1. Introduction

Plant pathogenic bacteria and fungi have had a significant impact on the agricultural economy, leading to numerous plant diseases and subsequent crop yield losses [1,2,3,4]. While synthetic pesticides have been a major source of crop protection since the 1960s, they have had detrimental effects on humans, animals, and the environment [5]. To surmount the harmful effects of chemical pesticides while ensuring crop protection, the search for and application of natural pesticides is imperative. Fungi, in this regard, have been found to be quite effective. Several endophytic fungal genera, such as *Penicillium* and *Aspergillus*, are known to contribute to plant protection through the production of diverse secondary metabolites [6,7]. Strobilurin-based commercial fungicides (inhibitors of cell respiration) produced by various Basidiomycetes fungi, have gained significant popularity with an increasing global market share [8]. Marine fungi, which have developed specific metabolic adaptation mechanisms to cope with the ever-changing ocean conditions (e.g., temperature, nutrients, and salinity) [9], are also receiving attention in crop protection. For example, the new benzopyranone (+)-(*2S*,*3R*,*4aR*)-altenuene produced by a marine *Alternaria* sp., exhibits robust antifungal activity against *Alternaria brassicicola*, the causative agent of black spot disease in a wide range of plant hosts [10].

Cultivation of microorganisms in standard laboratory conditions as axenic cultures often fails to unlock the maximum metabolic diversity of the organisms. This is due to the absence of environmental stimuli necessary for activating biosynthetic gene clusters (BGCs) in artificial laboratory conditions [11]. Genome sequencing has revealed that fungi harbor more BGCs predicted to encode more secondary metabolites than expressed [12,13]. The One Strain Many Compounds (OSMAC) approach has shown great promise in overcoming this hurdle and achieving enhanced chemical diversity [14]. It involves variations in culture parameters and conditions, e.g., media composition and temperature, to stimulate the expression of dormant BGCs to produce new metabolites. Culture regime, i.e., the use of solid or liquid medium under static or dynamic (i.e., shaking) culture conditions, also significantly influences microbial metabolism. Co-cultivation of two or more microorganisms in a confined environment has been shown to activate BGCs by inducing, e.g., competition for space and nutrients [15]. However, the selection of the most suitable microbial pairs and the outcome of the co-cultivation remain a challenge. Furthermore, co-cultivation can sometimes lead to the suppression of BGCs [16,17].

In a previous study [18], we systematically co-cultivated fungi sourced from a marine sediment sample, categorizing them as either strong or weak partners based on their anti-phytopathogenic potency. In brief, extracts that exhibited >80% inhibition (tested at 100 g/mL) against at least two phytopathogenic strains were categorized as “strong”, while extracts that exhibited <80% inhibition were categorized as “weak” partners. Various co-culture combinations were established between the weak and strong partners. The co-cultivation of *Plenodomus influorescens* (the strong partner) and *Pyrenochaeta nobilis* (the weak partner) on a solid Potato Dextrose Agar (PDA) medium revealed a competitive interaction involving the suppression of biosynthetic pathways and the concurrent upregulation of known metabolite(s). Chemical investigation of the co-culture afforded five polyketides: 12-membered macrolides dendrodolides E (**1**) and N (**2**), azaphilones spiciferinone (**3**) and 8*α*-hydroxy-spiciferinone (**4**), and a *bis*-naphtho-*γ*-pyrone cephalochromin (**5**). Although compounds **2** and **5** displayed potent anti-phytopathogenic activities against *Xanthomonas campestris* (causative agent of black rot in crucifers) and *Phytophthora infestans* (causative agent of potato blight disease), **1** could not be tested because of its low amount. Compared to the axenic culture of *P. influorescens*, the biosynthesis of compound **5** was enhanced approximately 12-fold in the co-culture [18].

The aim of the present study was to co-cultivate *P. influorescens* and *P. nobilis* in liquid culture conditions in order to assess whether it would lead to (i) an improved anti-phytopathogenic activity, (ii) BGC suppression or activation phenomenon, and (iii) higher titers of the purified (target) metabolites dendrodolide E (**1**) and N (**2**), cephalochromin (**5**), spiciferinone (**3**), and 8*α*-hydroxy-spiciferinone (**4**) obtained in the previous study [18], particularly those we were unable to test due to low quantities. Furthermore, while upscaling using liquid media is easier with high-capacity fermenters, the scalability of solid media cultures remains laborious and challenging. In an attempt to determine the optimal culture conditions for enhanced chemical diversity and higher anti-phytopathogenic activity, the fungal strains (mono- and co-cultures) were grown in three different liquid media, Potato Dextrose Broth (PDB), Sabouraud Dextrose Broth (SDB), and Czapek-Dox Broth (CDB) under both static (ST) and shaking (SH) culture conditions. The fungal mono- and co-culture extracts were comparatively analyzed by UPLC-MS/MS-based untargeted metabolomics using the Feature-Based Molecular Networking (FBMN) tool and simultaneously tested for their inhibitory activity against multiple phytopathogens. Finally, we compared the metabolite profile of the liquid co-culture extract (PDB, the most active broth) with that of the solid co-culture grown on PDA previously [18].

## 2. Results

### 2.1. Anti-Phytopathogenic Activity of the Fungal Extracts

Axenic cultures of *Pyrenochaeta nobilis* (Pyr), *Plenodomus influorescens* (Ple), and their co-culture (CC) were grown in three different liquid media, namely Potato Dextrose Broth (PDB), Sabouraud Dextrose Broth (SDB), and Czapek-Dox Broth (CDB) under static (ST) and shaking (SH) culture conditions. The selection of these media was based on their different carbon and nitrogen compositions. CDB utilizes sucrose, a disaccharide, as the carbon source, and sodium nitrate as the nitrogen source along with other essential elements [19]. SDB, on the other hand, incorporates glucose, a monosaccharide, as its carbon source and peptone as its nitrogen source [20]. PDB distinctively incorporates potato extract, a starch polymer, and the monomer dextrose, providing a rich medium for fungal growth [21]. Here, 21-day-old liquid fungal cultures were extracted with EtOAc and tested in vitro against six plant pathogens, namely *Phytophthora infestans*, *Xanthomonas campestris*, *Pseudomonas syringae*, *Erwinia amylovora*, *Ralstonia solanacearum*, and *Magnaporthe oryzae*. The static liquid cultures exhibited no activity against any of the tested phytopathogens (Table 1). The cultures from the shaking liquid regime displayed comparatively better potential against the phytopathogenic oomycete *P. infestans*, while all other test pathogens were unsusceptible to any of the fungal extracts. Only three extracts obtained under continuous shaking from the mono-cultures of *P. influorescens* in PDB and CBD media (Ple-PDB-SH and Ple-CDB-SH), as well as the co-culture of *P. influorescens* and *P. nobilis* in PDB medium (CC-PDB-SH), were active towards the pathogen *P. infestans* (Table 1). The highest activity was observed with the CC-PDB-SH extract (IC_50_ 21.7 µg/mL), which was three times more potent than the mono-culture Ple-PDB-SH (IC_50_ 69.5 µg/mL). Pyr-PDB-SH was inactive (IC_50_ > 100 µg/mL). The only other anti-phytopathogenic extract derived from the liquid regime was the monoculture of Ple-CDB-SH that exhibited an IC_50_ value of 30.5 µg/mL (Table 1).

The crude extract yields ranged from 0.6 mg to 16.8 mg per 100 mL broth (Table 1). The co-cultures generally afforded higher yields than their respective mono-cultures in all conditions. Extract yields from CDB were the lowest compared to all other media. As shown in Table 1, the static liquid culture regime generally resulted in lower extract yields for both axenic and co-cultures. The poor activity and low yield of extracts from static culture regimes can be attributed to poor microbial growth due to the lack of even oxygen circulation (aeration) throughout the broth.

### 2.2. Untargeted Metabolomics of Mono- and Co-Cultures under Shaking Conditions

**Figure 1:**
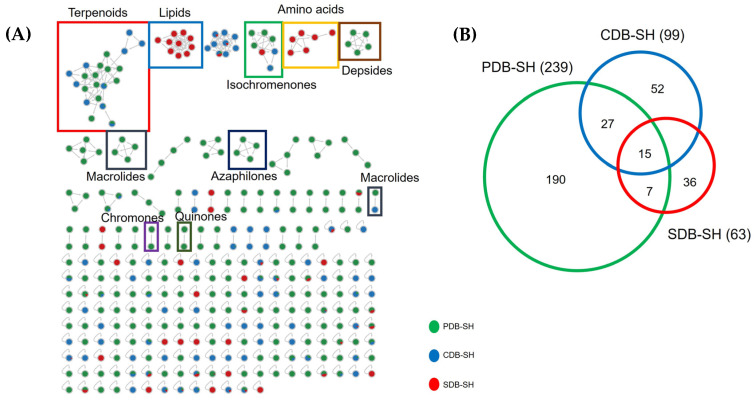
(**A**) The global molecular network of all mono- and co-cultures of *P*. *influorescens* and *P. nobilis* from all three media (PDB, SDB, and CDB) grown under shaking (SH) conditions. (**B**) A Venn diagram depicting the number of nodes generated in each mono- and co-culture of *P. influorescens* and *P. nobilis* in PDB-SH, CDB-SH, and SDB-SH media.

Based on the yields and activity profiles, a comparative untargeted metabolomics analysis using the Feature-Based Molecular Networking (FBMN) [22] tool was performed on the mono- and co-cultures of *P. influorescens* and *P. nobilis* obtained from the continuously shaken (SH) liquid PDB, CDB, and SDB media. The aim was to infer chemical variations and molecular families that may underlie the differential bioactivities observed. The inactive and low-yield static liquid culture extracts were excluded from further chemical analyses. The global MN of all the shaking liquid extracts comprised 332 nodes grouped into 46 clusters, of which 10 could be annotated to various molecular families (Figure 1A). Notably, the isochromenones were produced in all three media, while the clusters of depsides, azaphilones, quinones, and chromones were exclusive to PDB-SH extracts. Terpenoids and macrolides were largely expressed in PDB-SH and CDB-SH extracts of *P. influorescens* mono-culture and its co-culture with *P. nobilis*, while primary metabolites, e.g., amino acid derivatives and lipids, were found mainly in SDB-SH. The PDB-SH extracts displayed the largest number of nodes. A total of 239 nodes were produced, with 190 of them being exclusive to PDB-SH (Figure 1B). CDB-SH extract displayed 99 ions, while SDB-SH showed the least number of ions, 63 in total. The macrolide dendrodolide E (**1**) was exclusively produced in CDB-SH (Table S3), while all other target compounds were observed in the extracts of PDB-SH. Additionally, 20 clusters were exclusively produced in the mono- and co-cultures of PDB-SH, contributing to the high chemical diversity and bioactivity. Consequently, a molecular network was generated for Ple-PDB-SH, Pyr-PDB-SH, and CC-PDB-SH to comprehensively evaluate the metabolite production between the mono- and co-cultures (Section 2.3).

### 2.3. Molecular Network-Guided Analysis of Axenic and Co-Cultures in PDB-SH Medium

**Figure 2:**
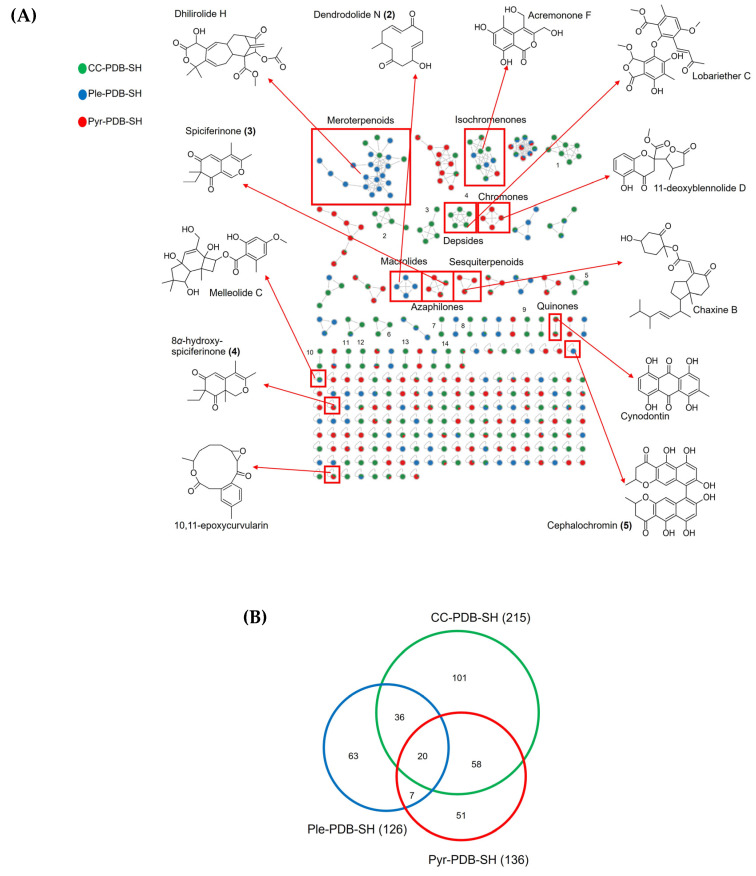
(**A**) FBMN of the extracts of the axenic and co-cultures, (**B**) Venn diagram depicting the number of nodes observed in the mono- and co-cultures of *P. influorescens* and *P. nobilis* in PDB-SH.

All mono- and co-culture extracts from PDB-SH medium were chosen for in-depth chemical analysis due to highest chemical diversity and bioactivity observed (Figure 1). This medium also presented the highest occurrence of target compounds, aligning with the observations from the solid PDA cultures [18]. Dereplication was carried out by multiple natural product databases such as GNPS library [23], Natural Product Atlas (npatlas, https://www.npatlas.org, accessed on 13 December 2023), and Dictionary of Natural Products (DNP, https://dnp.chemnetbase.com, accessed on 16 December 2023). A total of 12 compounds, belonging to 10 chemical classes were annotated based on their spectral similarities with natural products from various databases (Figure 2A, Table S2). Dhilirolide H (*m*/*z* 473.2152 [M + H]^+^) [24] was putatively identified in the meroterpenoid cluster, which is largely dominated by nodes originating from Ple-PDB-SH. Connected to dhilirolide H were some other isotopic ions that could not be identified and may represent new compounds. Nodes from the isochromenone cluster originated from all three cultures and displayed a low *m*/*z* range of 275 to 280 Da. Only one node could be annotated to acremonone F (*m*/*z* 275.0533, [M + Na]^+^) [25], and hence, the cluster was annotated as isochromenone. Other annotated compounds include the chromone 11-deoxyblennolide D (*m*/*z* 343.0791 [M + Na]^+^) [26] in Pyr-PDB-SH and CC-PDB-SH, quinone cynodontin (*m*/*z* 309.0377 [M + Na]^+^) [27] and terpenoid melleolide C (*m*/*z* 471.1991 [M + Na]^+^) [28] in Ple-PDB-SH, macrolide 10,11-epoxycurvularin (*m*/*z* 307.1183 [M + H]^+^) [29] and the sesquiterpenoid chaxine B (*m*/*z* 481.2933, [M + H]^+^) [30] in all cultures (Figure 2A).

With a focus on the target compounds, the macrolide dendrodolide N (**2**) (*m*/*z* 249.1202, [M + Na]^+^) [18] was identified in Ple-PDB-SH. Notably, all 4 nodes in the macrolide cluster showed similar *m*/*z* values and could represent isotopes of compound (**2**). Compound **1** was not expressed in PDB. Similar to the macrolides, the azaphilone cluster contained 4 nodes and was observed in both Pyr-PDB-SH and CC-PDB-SH. The node with *m*/*z* 233.1173 [M + H]^+^ was annotated as the azaphilone spiciferinone (**3**) [31]. The 8*α*-hydroxy analogue of spiciferinone (**4**) (*m*/*z* 251.128, [M + H]^+^) [18] was also annotated but as a single node. Unfortunately, it did not cluster with the azaphilone chemical family because of the slight change in fragmentation possibly induced by the hydroxylation. The *bis*-naphtho-*γ*-pyrone cephalochromin (**5**) (*m*/*z* 519.1281, [M + H]^+^) [32] was observed as a singleton in Ple-PDB-SH (Figure 2A).

Altogether, 14 clusters were exclusively induced in the co-culture extract (CC-PDB-SH, Figure 2A). One cluster was annotated as the depside family due to the putative identification of lobariether C (*m*/*z* 495.1264, [M + Na]^+^) [33]. Other co-culture-specific clusters remain unidentified and, hence, are potentially novel.

The FBMN method allowed for a semi-quantitative comparison of ions in different extracts [34,35,36], and thus, we could observe differential production of certain compounds in the mono-cultures versus the co-culture. For example, the amounts (measured by peak intensities) of dhilirolide H and cephalochromin (**5**) were higher in the axenic Ple-PDB-SH than in CC-PDB-SH, while compounds **3** and **4** were more abundant in Pyr-PDB-SH than in CC-PDB-SH. Moreover, the co-culture displayed a total of 101 exclusive nodes, while 63 and 51 nodes, respectively, were specific to *P. influorescens* and *P. nobilis* mono-cultures (Figure 2B). This result clearly indicates an increased chemical diversity in the fungal co-culture.

### 2.4. Analysis of the UPLC-MS Chromatograms of the Mono- and Co-Cultures in PDB-SH

**Figure 3:**
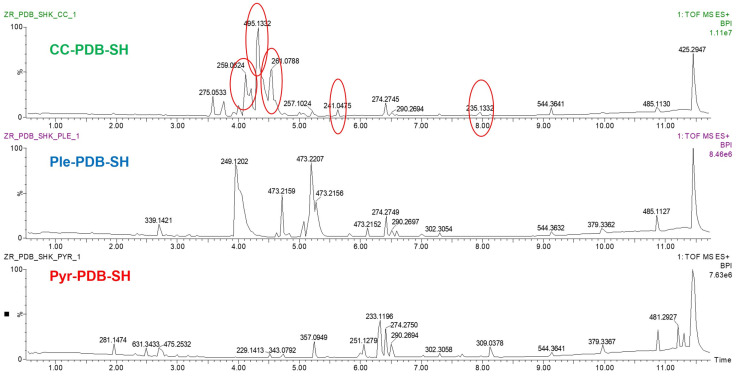
UPLC-MS chromatograms of the co-culture (**top**), and the mono-cultures of *Plenodomus influorescens* (**middle**) and *Pyrenochaeta nobilis* (**bottom**) grown in PDB-SH.

Comparison of the UPLC-MS chromatograms of the fungal co-cultures in PDB-SH medium (CC-PDB-SH) revealed patterns of both peak induction and suppression compared to the mono-cultures. In the co-culture, we observed five newly induced peaks at *t*_R_ 4.12 min (*m*/*z* 259.0624 [M + Na]^+^, C_12_H_13_O_5_, unannotated), *t*_R_ 4.33 min (*m*/*z* 495.1332 [M + H]^+^, C_19_H_27_O_15_, annotated as lobariether C), *t*_R_ 4.55 min (*m*/*z* 261.0788 [M + Na]^+^, C_12_H_15_O_5_, unannotated), *t*_R_ 5.63 min (*m*/*z* 241.0475 [M + Na]^+^, C_12_H_11_O_4_, unannotated), and *t*_R_ 7.97 min (*m*/*z* 235.1332 [M + H]^+^, C_14_H_19_O_3_, unannotated) (marked in Figure 3), while almost all other peaks observed in the respective liquid mono-cultures were significantly suppressed (Figure 3). These observations signify the upregulation of highly functional metabolite(s) in the co-culture mediating the competitive interaction while downregulating the other metabolites that are produced in their respective mono-cultures. Four of these significant peak ions remain unannotated because database searches for their predicted formulae returned hits that could not be verified by their MS^2^ fragments and biological sources, thus they may represent putative new compounds.

### 2.5. Comparison of Anti-Phytopathogenic Activity and Metabolomes of Co-Cultures in PDA and PDB Media

**Figure 4:**
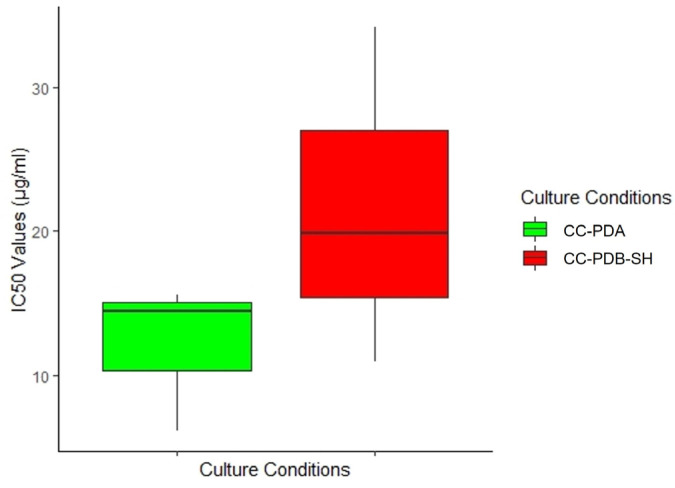
Comparison of the potency (IC_50_ values) of fungal co-cultures in liquid PDB-SH and solid PDA against *Phytophthora infestans*.

Previous work [18] analyzed the anti-phytopathogenic activity of the solid PDA co-culture extract of *P. influorescens* and *P. nobilis*. Here, we compare the bioactivity of the liquid co-culture (CC-PDB-SH) with the previously reported activity of the solid co-culture (CC-PDA) [18]. Both co-culture extracts were active against *P. infestans*, but the CC-PDA extract was more potent (IC_50_ 12.1 µg/mL) than the liquid co-culture extract CC-PDB-SH (IC_50_ 21.7 µg/mL). However, the difference was not statistically significant (*p* > 0.05) (Figure 4). While liquid CC-PDB-SH had a narrow-spectrum anti-phytopathogenic activity only against *P. infestans*, solid CC-PDA exerted broader-spectrum significantly inhibiting multiple phytopathogens, *P. infestans*, *M. oryzae*, and *X. campestris* [18].

**Figure 5:**
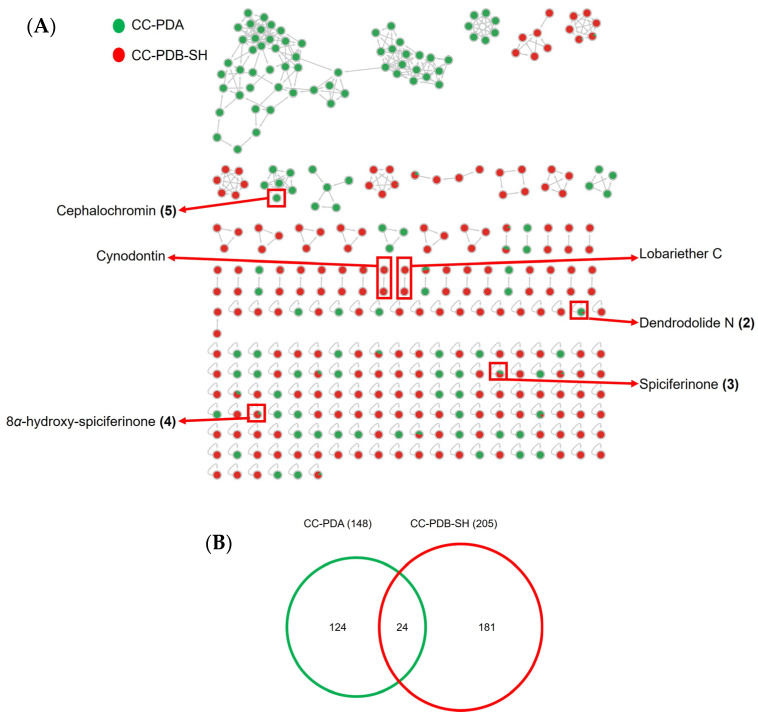
(**A**) FBMN of the co-culture extracts of *P. influorescens* and *P. nobilis* in PDA and PBD-SH extracts. (**B**) Venn diagram depicting the number of nodes observed in PDA and PDB-SH extracts.

Next, we compared the metabolome of the solid (PDA) and liquid (PDB-SH) medium co-culture in order to depict the impact of the culture regime and obtain hints on chemical constituents that may be contributing to differential activities. Thus, we generated an FBMN, and as shown in Figure 5B, the chemical diversity of the PDB-SH co-culture extract was higher (205 nodes) than the PDA co-culture (148 nodes). Although the co-cultures shared common nodes (24 nodes), numerous nodes were unique to individual extracts (124 in PDA and 181 in PDB-SH), highlighting the impact of the culture regime on fungal metabolism. In our previous work [18], we detected and purified dendrodolide N (**2**) and its derivative, dendrodolide E (**1**) (macrolides), spiciferinone (**3**), and 8*α*-hydroxy-spiciferinone (**4**) (azaphilones), and cephalochromin (**5**) (*bis*-naphtho-*γ*-pyrone) from the solid medium co-culture of *P. influorescens* and *P. nobilis* (PDA). In the current study, compound **1** was absent in the CC-PDB-SH (Figure 5A). This macrolide was expressed and identified only in the agar medium, but not under liquid culture conditions.

**Figure 6:**
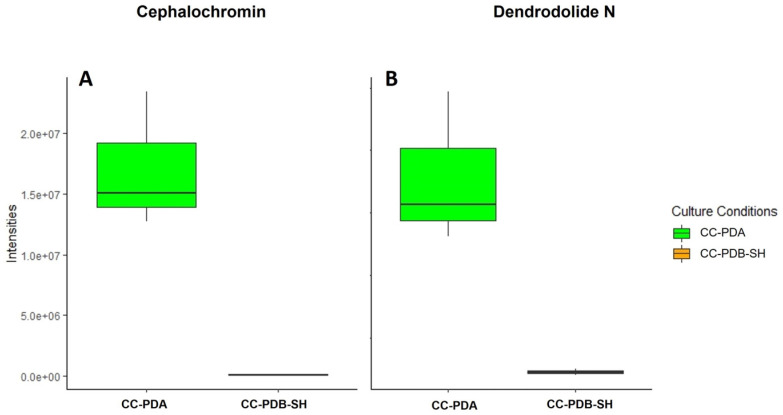
Abundance of (**A**) cephalochromin (**5**) and (**B**) dendrodolide N (**2**) in *P. nobilis* and *P. influorescens* co-cultures in PDA and PDB-SH.

**Figure 7:**
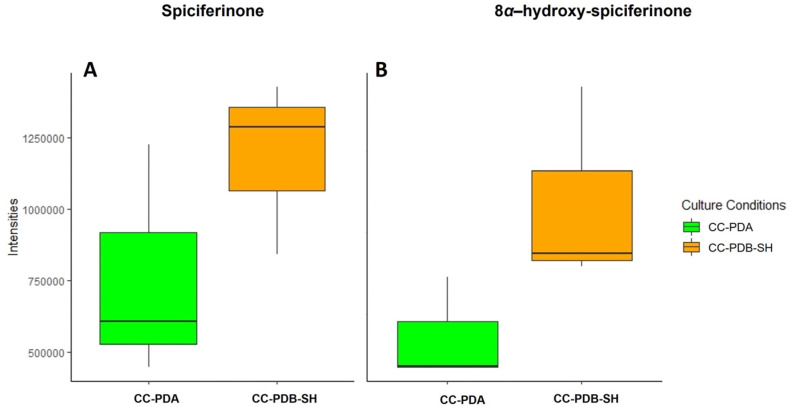
Abundance of (**A**) spiciferinone (**3**) and (**B**) 8*α*-hydroxy-spiciferinone (**4**) in *P. nobilis* and *P. influorescens* co-cultures in PDA and PDB-SH.

Moreover, the abundances of the target compounds, assessed by the average peak areas measured from the chromatograms, varied across different culture conditions. Compounds **2** and **5** were produced in significantly (*p* < 0.05) higher quantities in the solid (agar) culture (CC-PDA) (Figure 6), while compounds **3** and **4** were overexpressed in the liquid co-culture CC-PDB-SH (Figure 7).

## 3. Discussion

Both OSMAC and co-cultivation approaches are considered useful for the induction of microbial chemical diversity by stimulating the expression of cryptic biosynthetic gene clusters (BGCs) [37,38]. Based on our previous [18] and current observations, liquid cultivation mode (under shaking conditions) appears to offer more dynamism and greater chemical diversity compared to solid culture regime and static cultivation. This can be attributed to the more uniform distribution of oxygen in the culture medium, making it more available to microorganisms [39]. In our previous study [18], the metabolomes of *P. influorescens*, *P. nobilis*, and their co-culture on the solid PDA culture medium were comparatively analyzed. In contrast to the mono-cultures, the chemical profile of the co-culture displayed a marked reduction in the chemical diversity, indicating the suppression of BGCs in the co-culture. On the other hand, the distinction in the metabolic profile of the co-culture was particularly marked by the overexpression of cephalochromin (**5**). The co-culture also demonstrated enhanced bioactivity against two plant pathogens, *X. campestris* and *P. infestans* [18]. In the present study, co-cultivation was carried out in liquid media to determine the impact of the culture medium, especially on the production of the target compounds **1**–**5** in higher titers. This would allow better scalability in pilot plants while attempting to establish the most ideal culture conditions and parameters to produce the best anti-phytopathogenic activity. Additionally, the study aimed to assess the similarities and variations in BGC suppression/activation, resulting in different metabolite profiles in liquid PDB and solid PDA medium.

Of the selected liquid media (PDB, SDB, and CDB), PDB-SH provided the highest anti-phytopathogenic activity (when continuous shaking was applied). Potato dextrose represents one of the most commonly used growth media (in both liquid and solid regimes) to study colony morphology, biomass production, reproduction, survival, and production of natural products such as antibiotics [40]. The high extract yields obtained with PDB, in addition to their observed bioactivity and chemical profiles, highlight the significance of starch polymer and dextrose as carbon sources for fungal growth and secondary metabolism [41].

Two different extracts, Ple-PDB-SH and CC-PDB-SH, showed activity against *P. infestans* (IC_50_ 69.5 and 21.7 µg/mL, respectively). The molecular network-based annotation of compounds in PDB-SH revealed the induction of 14 exclusive clusters in the co-culture, which could have influenced its bioactivity. The induced compounds signify BGC upregulation in the co-culture. One such cluster is the putative depside cluster. This cluster contained the compound lobariether C, originally isolated from the fungus *Lobaria orientalis* [33]. Depsides are known to exhibit antitumor, antimicrobial, and antifouling activities [42,43]. In the chromatograms, lobariether C was annotated to a high-intensity peak ion at *t*_R_ 4.33 min, *m*/*z* 495.1332, [M + H]^+^ and is likely to exert anti-phytopathogenic effects as observed in the co-culture extract. PKS cluster activation has been previously associated with the biosynthesis of depsides in lichens [44], and the same phenomenon is likely to have stimulated depside production in our study as well. Cynodontin, a known quinone mycotoxin, has shown similar inhibitory effects against the phytopathogens *Sclerotinia minor*, *Sclerotinia sclerotiorum*, and *Botrytis cinerea* as commercial fungicides [45]. It was first isolated from *Curvularia* and *Drechslera* species [46], and later isolated from *Pyrenochaeta terrestris* [27]. The presence of the quinone cynodontin in the co-culture is likely to have resulted from the induction of iterative NR-PKSs [47], and may also be partly responsible for the observed activity against the tested phytopathogen *P. infestans*.

Additionally, the production of cephalochromin (**5**) in Ple-PDB-SH and CC-PDB-SH further supports the observed bioactivity, as this compound is known for its strong anti-phytopathogenic activity against *P. infestans*, *R. solanacearum*, and *X. campestris* [18]. *Bis*-naphtho-*γ*-pyrones from fungi are generally antibacterial agents with potent activities against *Mycobacterium tuberculosis* H37Rv, *Bacillus subtilis*, and *Staphylococcus aureus* [48]. Other known antimicrobial metabolites, namely the meroterpenoid dhilirolide H and the protoilludane sesquiterpenoid melleolide C, were putatively annotated in the extracts of Ple-PDB-SH and CC-PDB-SH. Dhilirolide H has been reported to exert inhibitory effects on the phytopathogen, *Trichoplusia ni* at low concentrations through significant feeding inhibition and sublethal developmental disruption [49], while melleolide C is a potent cytotoxic and antifungal agent inhibiting the growth of various fungi, including *Aspergillus nidulans*, *A. flavus*, and *Penicillium notatum* [28,50,51,52].

Except for the macrolide dendrodolide E (**1**), all the target compounds detected and identified in the solid PDA medium (compounds **2**–**5**) in the previous study [18] were identified in the liquid PDB medium in our study. However, the intensities of the biosynthesized target compounds varied greatly between the solid and liquid media, indicative of upregulation or downregulation of biosynthetic gene clusters responsible for synthesizing the target compounds. The extracts derived from PDB-SH displayed a larger number of compounds compared to those from PDA, aligning with the observation that dynamic liquid cultivation modes provide a broader spectrum of chemical diversity [39]. However, the anti-phytopathogenic activity was lower and limited only against *P. infestans*. Furthermore, although the PDB-SH contained a higher number of nodes in the MN, the production intensities of the macrolide dendrodolide N (**2**) and *bis*-naphtho-*γ*-pyrone cephalochromin (**5**) were significantly higher (*p* < 0.05) in PDA. This suggests the suppression of the respective BGCs in liquid broth. A previous study has shown that gene expression can vary depending on the cultivation conditions, resulting in varying production of metabolites in fungi [53]. Similarly, in our study, PKSs responsible for the production of macrolides and *bis*-naphtho-*γ*-pyrones were suppressed in the CC-PDB-SH while they were highly active in the CC-PDA. This phenomenon resulted in the higher production of dendrodolide N (**2**) and cephalochromin (**5**) in the CC-PDA. The presence of these compounds in high titers in CC-PDA aligns with the broader spectrum activity against the phytopathogens *P. infestans*, *M. oryzae*, and *X. campestris*. The macrolide dendrodolide N was reported for the first time in the previous study [18]. Macrolides are generally known to inhibit protein synthesis in bacteria by binding at the bacterial ribosome and obstructing the peptide chain formation [54], while 12-membered macrolide derivatives exhibit various bioactivities as well [55]. The pronounced impact of the overexpression of cephalochromin (**5**) in CC-PDA cannot be overemphasized, given its potent activity exhibited against *P. infestans* and *X. campestris* in previous studies [18,48]. These findings highlight the potential importance of cephalochromin (**5**) as a key contributor to the observed inhibitory effect of the CC-PDA extract. The strong activity demonstrated by cephalochromin and dendrodolides against these phytopathogens suggests their potential as promising candidates for further exploration and development for plant disease management.

Liquid cultivation did not, however, offer high promise for upscaling as the production of cephalochromin (**5**) and dendrodolide N (**2**) was in low titers in the CC-PDB-SH. On the other hand, the target azaphilones, spiciferinone (**3**), and 8*α*-hydroxy-spiciferinone (**4**) were produced in higher amounts in the CC-PDB-SH than in the CC-PDA, indicating the upregulation of the respective PKS genes in the liquid broth. Although these compounds were inactive against the panel of phytopathogens in the previous study [18], their enhancement in the CC-PDB-SH will facilitate testing against a broader panel of phytopathogens or other pathogens following upscaling. In the case of CC-PDB-SH, due to the detection of a relatively low concentration of cephalochromin (**5**), it is unclear whether it is responsible for the observed activity against *P. infestans*. It is possible that lobariether C and cynodontin, compounds with well-known fungicidal potential [42,43,45] and which were also observed as intense induced peaks in the chromatogram of CC-PDB-SH, may contribute to the activity. However, they were not biosynthesized in CC-PDA indicating suppression of the respective genes in the solid medium. A similar scenario was observed for the marine-derived *Streptomyces* sp. USC-633 [56]. When cultivated using a solid agar medium, the extract displayed bioactivity against the human pathogens *Enterococcus faecalis* and *Escherichia coli* ATCC 13706, whereas the broth medium extract only displayed activity against the pathogen *E. coli* ATCC 13706 [56]. Further investigations revealed distinct metabolite profiles between agar and broth cultures, suggesting differential metabolite implications in the observed bioactivity profiles [56].

In general, the two co-cultures (CC-PDA and CC-PDB-SH) displayed pronounced differences in their metabolic profiles and bioactivities, signifying the impact of varying culture regimes (solid and liquid) on the metabolic diversity of microorganisms. While the bioactivity in CC-PDA was highlighted by the overexpression of cephalochromin (**5**), the bioactivity in CC-PDB-SH was primarily driven by the biosynthesis of cynodontin and lobariether C. Our study further demonstrates the impact of the OSMAC approach in microbial biodiscovery.

## 4. Materials and Methods

### 4.1. Fungal Cultures and Extraction

*Pyrenochaeta nobilis* and *Plenodomus influorescens* were isolated from sediment samples collected in the Baltic Sea environment, specifically from the Windebyer Noor in Schleswig-Holstein, Germany, as previously described [57]. Briefly, sample obtained from sediment coring using 50 mL sterile Falcon tube was transferred (about 0.1 g) into a 2 mL innuSPEED lysis tube type S containing 0.4–0.6 mm ceramic beads (Analytik Jena, Jena, Germany). After adding 600 μL of sterile 0.9% saline with gentle agitation, sample was plated on different cultivation media [57]. *Pyrenochaeta nobilis* and *Plenodomus influorescens* were isolated on Glucose Peptone Yeast and Hastings media, respectively [57].

The fungal strains were maintained on solid PDA (20 g of glucose, 4 g of potato infusion, and 7.5 g of Bacto Agar in 1-liter dH_2_O) medium at 22 °C in the dark. The mycelial discs (5 mm diameter) from the respective agar cultures were transferred into 50 mL of PDB (20 g of glucose, 4 g of potato infusion in 1-liter dH_2_O), SDB (10 g of Bacto Peptone and 20 g of glucose in 1-liter dH_2_O), and CDB (35 g of Czapek-Dox containing 30 g of saccharose, 3 g of NaNO_3_, 1 g of dipotassium phosphate, 0.5 g of MgSO_4_, 0.5 g of KCl, and 0.01 g of FeSO_4_ in 1-liter dH_2_O) as precultures. Precultures were incubated in the dark at 22 °C on a rotary shaker (120 rpm) for 7 days. For axenic cultures, 1 mL of each strain was inoculated from their pre-cultures into their respective broths (PDB, SDB, and CDB), each containing 100 mL of broth in 300 mL Erlenmeyer flasks. For co-cultivation, 1 mL of each strain was transferred from their pre-cultures into 100 mL of broths in 300 mL Erlenmeyer flasks. All the cultures were grown in triplicate and incubated in the dark at 22 °C on a rotary shaker for 21 days to obtain the shaking cultures (SH). The same procedure was applied to the static cultures (ST), except the broths were incubated in the dark at 22 °C without shaking for 21 days.

After 21 days of incubation, cultures were homogenized with an Ultra-Turrax (IKA-Werke, Staufen, Germany) at 19,000 rpm, extracted with EtOAc (PESTINORM, VWR Chemicals, Leuven, Belgium) (2 × 100 mL) in separatory funnels (1000 mL) and washed twice with 100 mL of Milli-Q^®^ water (Arium^®^ Lab water systems, Sartorius) to remove salts and water-soluble compounds. EtOAc extracts were then evaporated to dryness on a rotary evaporator under reduced pressure (200 bar, 150 rpm at 40 °C). The dried crude extracts were re-dissolved in ULC/MS grade MeOH and transferred to 4 mL pre-weighed storage vials through 13 mm syringe filters with 0.2 µm PTFE membranes (VWR International, Darmstadt, Germany). The extracts in the vials were dried under nitrogen. The whole procedure was repeated for all replicates from all media and culture conditions (shaking and static) as well as media blanks. Obtained extracts underwent additional purification using solid phase extraction (SPE). Extracts were individually applied onto C_18_ (3 mL/50 mg) cartridges (Macherey-Nagel, Duren, Germany) and firstly eluted with water (10 mL), then with MeOH (10 mL). The methanolic phase was vacuum-dried to afford the crude extracts used in chemical and bioactivity profiling.

### 4.2. UPLC-QToF-MS Analysis

Chromatograms were acquired on the UPLC I-Class System coupled to a Xevo G2-XS QToF Mass Spectrometer (Waters^®^, Milford, MA, USA) at a concentration of 0.1 mg/mL of extract. Sample separation was achieved on an Acquity UPLC HSS T3 column (High Strength Silica C_18_, 1.8 µm, 100 × 2.1 mm, Waters^®^) at 40 °C and an injection volume of 2 µL, using a binary solvent system composed of mobile phase A (MilliQ^®^-water with 0.1% formic acid (ULC/MS grade)) and phase B (acetonitrile (MeCN, ULC/MS grade, Biosolve BV, Dieuze, France) with 0.1% formic acid) in a linear gradient. The mobile phase was pumped at a flow rate of 0.3 mL/min with the following gradient: 1% to 99% A for 11.5 min, 0% A for 1 min, and 99% A until minute 15. MS and MS/MS spectra were acquired in a data-dependent analysis (DDA) mode with electrospray ionization (ESI) source in the positive mode using the following parameters: mass range of *m*/*z* 50–1200 Da, capillary voltage of 0.8 kV, cone and desolvation gas flow of 50 and 1200 L/h, respectively, source temperature at 150 °C, and desolvation temperature at 550 °C, with sampling cone and source offset at 40 and 80, respectively. Collision energy (CE) was ramped: low CE from 6–60 eV to high CE of 9–80 eV. As controls, solvent (MeOH) and extracts of non-inoculated media (media blank) were injected under the same conditions.

### 4.3. Molecular Networking

Data obtained from the UPLC-MS/MS system were converted to mzXML format using Proteo Wizard msconvert (version 3.0.22074 64-bit, Vanderbilt University, Nashville, TN, USA) [58]. The converted spectra were then processed in MZmine (version 2.33) [59] using the following modules: Mass detection (RT 1.0–11.5 min, centroid); Chromatogram builder (MS level 1; minimum height 2.0 × 104; minimum time span 0.01 min; *m*/*z* tolerance 20 ppm); Chromatogram deconvolution (Algorithm Baseline cut-off; peak duration 0.0–0.5 min; MS^2^ pairing *m*/*z* range 0.5 min, MS2 pairing RT range 0.2 min); Isotopic peaks grouper (*m*/*z* tolerance 20 ppm; RT tolerance 0.3 min); Data alignment (Join aligner; *m*/*z* tolerance 20 ppm; RT tolerance 0.5 min); and Peak list filtering (RT 1.0–11.5 min). The processed data were uploaded onto the Global Natural Products Social Molecular Networking (GNPS) platform and analyzed using the molecular networking workflow (http://gnps.ucsd.edu, accessed on 3 July 2022) [60,61]. For the generation of the molecular networks, MS/MS spectra were window-filtered by choosing only the top six peaks in the +/−50 Da window throughout the spectra. The data were clustered with MS-Cluster with a parent mass tolerance and MS/MS fragment ion tolerance of 0.02 Da. Further, consensus spectra that contained less than two spectra were discarded. A network was then created where edges were filtered to have a cosine score above 0.7 and more than six matched peaks. Furthermore, spectra in the network were then searched against GNPS spectral libraries, and only matches with a score above 0.7 and at least 6 matched peaks were kept. The molecular networking data were visualized in Cytoscape 3.9.0. [62] program using a ‘directed’ style. All compounds (nodes) originating from the media and solvent control (MeOH) were deleted from the original network enabling visualization of metabolites coming from mono- and co-cultures.

Manual annotation of peak ions was performed using MassLynx^®^ version 4.1. by searching their predicted molecular formula against databases such us NPAtlas (https://www.npatlas.org/, accessed on 13 December 2023), COCONUT (https://coconut.naturalproducts.net/, accessed on 10 December 2023), LOTUS (https://lotus.naturalproducts.net/, accessed on 12 December 2023), Dictionary of Natural Products (https://dnp.chemnetbase.com/, accessed on 16 December 2023), and PubChem (https://pubchem.ncbi.nlm.nih.gov/, accessed on 15 December 2023). All hits were validated based on their biological source and fragmentation patterns using the CFM-ID web server (https://cfmid.wishartlab.com/, accessed on 17 December 2023).

### 4.4. Bioassays

All extracts were tested in triplicate against a panel of plant pathogens including *Erwinia amylovora*, *Pseudomonas syringae*, *Ralstonia solanacearum*, *Xanthomonas campestris*, *Magnaporthe oryzae*, and *Phytophthora infestans* using a broth dilution approach in a 96-well microplate as described before [57]. The assay incubation time for bacterial phytopathogens was 9 h (shaking at 200 rpm) at 28 °C, whereas the fungal phytopathogens were incubated for 96 h (shaking at 200 rpm) at 22 °C. The crude extracts were dissolved in DMSO and added to the test organisms in 96-well microplates to make an effective test concentration of 100 µg/mL and 0.5% (*v*/*v*) DMSO (final concentration). Chloramphenicol was used as the positive control against *P. syringae*, *X. campestris*, and *E. amylovora*, while tetracycline, cycloheximide, and nystatin served as positive controls against *R. solanacearum*, *P. infenstans*, and *M. oryzae*, respectively. The growth media (PDB, SDB, and CDB) and 0.5% (*v*/*v*) DMSO were tested as negative controls. Absorbances were measured at 600 nm using an Infinite M200 plate reader (TECAN Deutschland GmbH, Crailsheim, Germany) before and after incubation, and % inhibition values were computed. The IC_50_ values of the active crude extracts (that inhibit more than 50% of the pathogen growth at 100 µg/mL test concentration) were calculated using the Microsoft Excel (version 2312) program.

## 5. Conclusions

In summary, we examined the chemical and anti-phytopathogenic activity of marine-sediment derived fungi *Plenodomus influorescens* and *Pyrenochaeta nobilis* grown in three liquid media with or without shaking. Our goal was to improve culture conditions and parameters to enhance the bioactivity and expression of (bioactive) secondary metabolites by probing different culture regime and media. Concurrently, we compared our findings from the liquid broth (PDB-SH) with those from the solid agar medium as reported in our previous study [18]. Notably, significant variations in BGC regulation were observed between the PDB and PDA extracts, leading to differences in the biosynthesis of compounds and subsequently impacting the bioactivity of the extracts. Among all the extracts from liquid broths, the co-culture of *P. influorescens* and *P. nobilis* in PDB-SH (CC-PDB-SH) exhibited the highest anti-phytopathogenic activity. However, this bioactivity was still lower than that observed in CC-PDA [18], primarily due to the observed disparity in the abundance and the diversity of the biosynthesized secondary metabolites. This study demonstrates the importance of culture regime and culture conditions for chemical and bioactivity profile of fungal co-cultures. It also demonstrates the challenges associated with co-cultivation experiments for optimization and improvement of bioactive compound production. Our future efforts will include further variations, e.g., further tweaking the culture parameters (e.g., addition of nutrients, minerals, epigenetic modifiers, changing the pH, temperature, etc.) to ensure higher titers of the target compounds and enhanced bioactivity in liquid media.

## Figures and Tables

**Table 1:** Anti-phytopathogenic activity (against *Phytophthora infestans*) and extract yields of mono- and co-cultures (CC) of *P. influorescens* (Ple) and *P. nobilis* (Pyr) in three liquid media (PDB, CDB, and SDB) under shaking (SH) and static (ST) conditions. * The data from our previous work [18].

Code	Medium	Condition	Yield (mg)	IC_50_ (µg/mL)
CC-PDB-SH	PDB	Shaking	13.4	**21.7**
Pyr-PDB-SH			4.7	>100
Ple-PDB-SH			16.8	**69.5**
CC-PDB-ST		Static	4.7	>100
Pyr-PDB-ST			2.7	>100
Ple-PDB-ST			0.8	>100
CC-SDB-SH	SDB	Shaking	16.6	>100
Pyr-SDB-SH			7.9	>100
Ple-SDB-SH			6.4	>100
CC-SDB-ST		Static	6.9	>100
Pyr-SDB-ST			5.4	>100
Ple-SDB-ST			3.9	>100
CC-CDB-SH	CDB	Shaking	4.2	>100
Pyr-CDB-SH			5.5	>100
Ple-CDB-SH			3.7	**30.5**
CC-CDB-ST		Static	2.4	>100
Pyr-CDB-ST			1.9	>100
Ple-CDB-ST			0.6	>100
CC-PDA *	PDA		17.9 *	12.1 *
Pyr-PDA *			9.3 *	39.6 *
Ple-PDA *			65.4 *	8.6 *

## Data Availability

The molecular networking jobs on GNPS can be found at https://gnps.ucsd.edu/ProteoSAFe/status.jsp?task=ea4448e252284f89ba8e7cfe1717b627 (accessed on 3 July 2022) (*P. nobilis* monocultures, *P. influorescens* monocultures and their co-cultures grown in Czapek-Dox Broth under shaking conditions), https://gnps.ucsd.edu/ProteoSAFe/status.jsp?task=eedfd7533e0e4170bf0118a2e3c140ec (accessed on 3 July 2022) (*P. nobilis* monocultures, *P. influorescens* monocultures and their co-cultures grown in Potato Dextrose Broth under shaking conditions) and https://gnps.ucsd.edu/ProteoSAFe/status.jsp?task=52958df4cb9e4c3494db0c6292de7e8f (accessed on 10 April 2022) (Comparison between co-cultures of *P. nobilis* and *P. influorescens* grown in Potato Dextrose Agar and Potato Dextrose Broth).

## Supplementary Materials

The following supporting information can be downloaded at: https://www.mdpi.com/article/10.3390/md22020066/s1, Table S1: Bioactivity (% inhibition at 100 μg/mL) of different culture extracts against plant pathogens; Table S2: Putative annotations of fungal metabolites in PDB-SH; Table S3: Putative annotations of fungal metabolites in CDB-SH.
