# Supplementary material for: The Impact of the Culture Regime on the Metabolome and Anti-Phytopathogenic Activity of Marine Fungal Co-Cultures
Source: Mar Drugs. 2024 Jan 27;22(2):66. doi: 10.3390/md22020066 (PMC10890130; doi:10.3390/md22020066)
Supplement: Supplementary file 1 [file marinedrugs-22-00066-s001.zip › marinedrugs-2813912-supplementary.pdf]

Supporting Information

The Impact of the Culture Regime on the Metabolome and Anti-Phytopathogenic Activity of Marine Fungal Co-Cultures

Mohammed Zawad Reza [1], Ernest Oppong-Danquah [1] and Deniz Tasdemir [1,2,*]

[1] GEOMAR Centre for Marine Biotechnology (GEOMAR-Biotech), Research Unit Marine Natural Product Chemistry, GEOMAR Helmholtz Centre for Ocean Research Kiel, Wischhofstrasse 1-3, 24148 Kiel, Germany; zawadreza@gmail.com (M.Z.R.); eoppong-danquah@geomar.de (E.O.-D.)

[2] Faculty of Mathematics and Natural Sciences, Kiel University, Christian-Albrechts-Platz 4, 24118 Kiel, Germany

[*] Correspondence: dtasdemir@geomar.de; Tel.: +49-431-600-4430

List of Tables **Page**

Table S1. Bioactivity (% inhibition at 100 μg/mL) of different culture extracts against plant pathogens 2

Table S2. Putative annotation of fungal metabolites in PDB-SH medium 4

Table S3. Putative annotation of fungal metabolites in CDB-SH medium 17

Table S1. Bioactivity (% inhibition at 100 μg/mL) of different fungal media extracts against plant pathogens.

Pss = *Pseudomonas syringae*, Xc = *Xanthomonas campestris*, Ea = *Erwinia amylovora*, Rs = *Ralstonia solanacearum*, Pi = *Phytophthora infenstans*, Mo = *Magnaporthe oryzae*, Ple = *P. influorescens* monoculture, Pyr = *P. nobilis* monoculture, and CC = Co-culture. Positive controls: chloramphenicol for *P. syringae*, *X. campestris*, and *E. amylovora*, tetracycline for *R. solanacearum*, Cycloheximid for *P. infenstans*, Nystatin for *M. oryzae*. (-) indicates no activity.

Inhibition %

Extracts	Culture Conditions	Phytopathogenic Bacteria				Phytopathogenic Fungi	
		Pss	Xc	Ea	Rs	Pi	Mo
		100 μg/mL	100 μg/mL	100 μg/mL	100 μg/mL	100 μg/mL	100 μg/mL
Negative Control	Media Blank	20	-	-		-	-
Ple	PDB (Shaking)	-	10	-	-	76	-
CC	PDB (Shaking)	-	-	-	-	98	47
Pyr	PDB (Shaking)	-	-	-	-	17	-
Ple	PDB (Static)	-	-	-	-	-	-
CC	PDB (Static)	-	-	-	-	-	-
Pyr	PDB (Static)	-	-	-	-	7	-
Ple	CZA (Shaking)	-	-	-	-	96	-
CC	CZA (Shaking)	-	-	-	-	-	-
Pyr	CZA (Shaking)	-	-	-	-	-	-
Ple	CZA (Static)	-	-	-	-	18	-
CC	CZA (Static)	-	-	-	-	-	-
Pyr	CZA (Static)	-	-	-	-	-	-
Ple	SDB (Shaking)	-	22	-	-	33	-
CC	SDB (Shaking)	8	12	-	-	15	-
Pyr	SDB (Shaking)	-	19	-	-	9	-

Ple	SDB (Static)	-	-	-	-	-	-
CC	SDB (Static)	-	-	-	-	15	-
Pyr	SDB (Static)	-	-	-	-	-	-
Positive Control	-	97	92	91	77	93	97

Table S2. Putative annotations of fungal metabolites in PDB-SH. Dereplication of the fungal extracts using GNPS library search and manual dereplication based on NP Atlas, Coconut, and Lotus databases. Annotation considered the predicted molecular formula, retention time, *m/z*, ppm, fragmentation pattern, and biological source. PDB = Potato Dextrose Broth, SHK = Shaking, CC = Co-culture, PYR = *Pyrenochaeta nobilis* monoculture, and PLE = *Plenodomus influorescens* monoculture, N/A = Not Available.

Organism	Compound	Molecular Formula	Retention Time (Min)	*m/z* $[M+H]^+$	ppm	MS/MS $[M+H]^+$	Na+ Adduct	Biological Source	Bioactivity	Reference
PDB-SHK-CC	Acremonone F	$C_{12}H_{12}O_6$	3.6	253.071	-0.8	137.0242; 147.0068 151.0027; 165.0177 209.0431; 253.0677	275.0533	*Acremonium* sp. PSU-MA70	N/A	Rukachaisirikul et al., 2012 [1]
PDB-SHK-CC	Unidentified	$C_{13}H_{17}O_6$	3.8	269.102	-1.9	259.058; 233.0416	291.0842			
PDB-SHK-CC	Unidentified	$C_{12}H_{13}O_5$	4.0	237.0753	-4.2	215.0311; 155.0025	259.0579			
PDB-SHK-CC	Unidentified	$C_{12}H_{13}O_5$	4.1	237.0765	0.8	237.0756; 191.0706; 219.065; 163.0751; 135.0804	259.0624			
PDB-SHK-CC	Unidentified	$C_{12}H_{13}O_5$	4.2	237.0761	-0.8	237.0764; 219.0654; 193.0495; 163.0748	259.0592			
PDB-SHK-CC	Unidentified	$C_{19}H_{27}O_{15}$	4.3	495.1332	-3.6	433.0811; 425.0809; 407.0673	N/A			
PDB-SHK-CC	Unidentified	$C_{12}H_{15}O_5$	4.5	239.092	0.4	239.0911; 221.0808; 195.0649; 149.0599; 95.0502	261.0788			
PDB-SHK-CC	Unidentified	$C_{12}H_{13}O_5$	4.9	237.0759	-1.7	236.0908; 153.0542; 126.0552; 111.0445	259.0579			
PDB-SHK-CC	Unidentified	$C_{12}H_{17}O_6$	5.2	257.1024	-0.4	195.0652; 213.0754; 221.0819; 239.0918; 259.0571	279.0936			
PDB-SHK-CC	Unidentified	$C_{12}H_{11}O_4$	5.6	219.0653	-1.8	219.065; 177.0181; 145.0635; 95.9728	241.0475			
PDB-SHK-CC	Unidentified	$C_{12}H_{13}O_4$	5.9	221.0809	-2.3	204.1086	243.0631			

Organism	Compound	Molecular Formula	Retention Time (Min)	*m/z* [M+H]+	ppm	MS/MS [M+H]+	Na+ Adduct	Biological Source	Bioactivity	Reference
PDB-SHK-CC	Spiciferinone	$C_{14}H_{16}O_3$	6.3	233.1175	-1.3	205.0858; 233.1169; 176.0828	N/A	*Cochliobolus spicifer*	N/A	Nakajima et al., 1992[2]
PDB-SHK-CC	Unidentified	$C_{14}H_{19}O_3$	7.7	235.1333	-0.4	235.1324; 217.1239; 142.0775; 157.101; 175.1116; 185.096; 203.1056	257.1153			
PDB-SHK-CC	Unidentified	$C_{14}H_{19}O_3$	7.9	235.1332	-0.9	235.1325; 185.0963; 175.1119; 157.1008; 142.078; 119.0863; 105.0704	257.1151			
PDB-SHK-CC	Cynodontin	$C_{15}H_{10}O_6$	8.1	287.0554	-0.7	97.971; 113.9638; 141.9718; 156.8904	309.0377	*Pyrenochaeta terrestris*	Antifungal	Kurobane et al., 1979[3]
PDB-SHK-CC	4,4,6a,10,12b,14b-hexamethyl-1,2,3,4,4a,5,6,6a,6b,7,8,12b,13,14,14a,14b-hexadecahydropicene	$C_{28}H_{43}$	9.9	379.3362	-0.8	379.3365; 337.292; 323.2768; 309.2575	401.839	N/A	N/A	N/A
PDB-SHK-CC	Lobariether C	$C_{24}H_{24}O_{10}$	5.0	473.1445	-0.6	163.0764; 193.0487; 219.0651; 237.0755; 291.0491; 399.0666	495.1267	*Lobaria orientalis*	Antifungal	Nguyen et al., 2017[4]
PDB-SHK-CC	Unidentified	$C_{12}H_{17}O_6$	5.2	257.1022	-1.2	121.0651; 177.054; 195.0652; 213.0752	279.0934			
PDB-SHK-CC	Unidentified	$C_{12}H_{11}O_4$	5.6	219.0649	-3.7		241.0475			
PDB-SHK-PYR	Unidentified	$C_{37}H_{53}O_5S$	2.5	609.361	-0.7	254.1517; 113.0613; 195.1134; 271.1753; 436.3381; 497.3048	631.3432			
PDB-SHK-PYR	11-deoxyblennolide D	$C_{16}H_{16}O_7$	4.7	321.0971	-0.9	247.0892; 258.0813; 275.0885; 288.0661; 303.0874	343.0791	*Setophoma terrestris*	Antitumor	El-Elimat et at., 2015[5]

Organism	Compound	Molecular Formula	Retention Time (Min)	*m/z* $[M+H]^+$	ppm	MS/MS $[M+H]^+$	Na+ Adduct	Biological Source	Bioactivity	Reference
PDB-SHK-PYR	Unidentified	$C_{17}H_{18}O_7$	5.2	335.1127	-1.2	325.0681; 297.0845; 283.0555	357.0949			
PDB-SHK-PYR	8a-hydroxy-spiciferinone	$C_{14}H_{18}O_4$	5.9	251.128	-1.2	251.1264; 233.1166; 226.9238; 215.1065; 207.1335	273.1665	*Pyrenochaeta nobilis*	N/A	Oppong-Danquah et al., 2020[6]
PDB-SHK-PYR	Spiciferinone	$C_{14}H_{16}O_3$	6.3	233.1175	-1.3	205.0858; 233.1169; 176.0828	N/A	*Cochliobolus spicifer*	N/A	Nakajima et al., 1992[2]
PDB-SHK-PYR	10,11-epoxycurvularin	$C_{16}H_{18}O_6$	7.6	307.1183	0.3	307.1173; 289.1073; 290.1101	329.2283	*Penicillium* sp.	N/A	Aly et al., 2011[7]
PDB-SHK-PYR	Unidentified	$C_{14}H_{19}O_3$	7.9	235.1332	-0.9	235.1334; 175.1115; 185.0958; 189.1273; 193.1223; 203.106	257.1151			
PDB-SHK-PYR	Cynodontin	$C_{15}H_{10}O_6$	8.1	287.0554	-0.7	97.971; 113.9638; 141.9718; 156.8904	309.0377	*Pyrenochaeta terrestris*	Antifungal	Kurobane et al., 1979[3]
PDB-SHK-PYR	Unidentified	$C_{28}H_{43}$	9.9	379.3366	0.3	379.3362; 404.3006; 364.3156; 337.2886; 323.2744	401.8401	N/A	N/A	N/A
PDB-SHK-PYR	Chaxine B	$C_{28}H_{42}O_5$	11.2	481.2927	-0.6	481.2922; 463.2804; 423.2214	N/A	*Agrocybe chaxingu*	N/A	Choi et al., 2009[8]
PDB-SHK-PLE	Dendrodolide N	$C_{12}H_{18}O_4$	3.9	227.1281	-0.9	192.9799; 207.0689; 187.1098; 175.0777	249.1202	*Plenodomus influorescens*	Antifungal	Oppong-Danquah et al., 2020[6]
PDB-SHK-PLE	Melleolide C	$C_{24}H_{32}O_8$	4.6	449.203	1.6	171.8614; 247.0946; 348.2002; 409.1969	471.1991	*Armillaria mellae*	Antibacterial	Arnone et al., 1986[9]
PDB-SHK-PLE	Dhilirolide H	$C_{26}H_{32}O_8$	4.7	473.2159	-3.4	473.215; 455.1935; 431.2032; 411.2138; 403.1732; 385.1987; 369.206	N/A	*Penicillium purpurogenum*	N/A	Centko et al., 2014[10]
PDB-SHK-PLE	Unidentified	$C_{24}H_{35}O_8$	5.1	451.2326	-1.3	413.1938; 377.1559; 265.1049; 249.1095; 151.0357	473.2207			

Organism	Compound	Molecular Formula	Retention Time (Min)	*m/z* $[M+H]^+$	ppm	MS/MS $[M+H]^+$	Na+ Adduct	Biological Source	Bioactivity	Reference
PDB-SHK-PLE	Unidentified	$C_{28}H_{43}$	9.9	379.3362	-0.8	379.3357; 337.2874	401.8374			
PDB-SHK-PLE	Prenylcandidusin B	$C_{27}H_{28}O_6$	4.6	449.1985	4.7		471.1996	*Aspergillus taichungensis* ZHN-7-07	N/A	Cai et al., 2011[11]

Table S3. Putative annotation of fungal metabolites in CDB-SH. Dereplication of the fungal extracts using GNPS library search and manual dereplication based on NP Atlas, Coconut, and Lotus databases. Annotation considered the predicted molecular formula, retention time, *m/z*, ppm, fragmentation pattern, and biological source. CZA = Czapek-Dox Broth, SHK = Shaking, CC = Co-culture, PYR = *Pyrenochaeta nobilis* monoculture and PLE = *Plenodomus influorescens* monoculture, N/A = Not Available.

Organism	Compound	Molecular Formula	Retention Time (Min)	*m/z* $[M+H]^+$	ppm	MS/MS $[M+H]^+$	Na+ Adduct	Biological Source	Bioactivity	Reference
CZA-SHK-CC	Isobemisiose	$C_{18}H_{32}O_{16}Na$	1.1	505.769	1.1		527.1594	*Aspergillus fischeri*	N/A	Wyatt et al., 2015[12]
CZA-SHK-CC	Unidentified	$C_{38}H_{42}O_{17}$	2.6	771.249	-1.3	765.2364; 583.117; 541.1464;430.0392; 402.0453; 345.0243; 276.0027; 149.0684	793.2313			
CZA-SHK-CC	Clavilactone H	$C_{17}H_{18}O_4$	7.4	287.1282	-0.3	287.1277; 269.1182; 259.1328; 241.123; 219.0651	N/A	*Clitocybe clavipes*	Cytotoxicity	Sun et al., 2019[13]
CZA-SHK-CC	Unidentified	$C_{17}H_{25}O_{11}$	2.5	405.1382	-3.7	203.0771; 245.1254; 247.095; 283.1041; 300.1322; 301.1173; 319.1272; 326.8055; 343.1421; 345.107; 362.1308; 363.1334	427.1573			

Organism	Compound	Molecular Formula	Retention Time (Min)	*m/z* $[M+H]^+$	ppm	MS/MS $[M+H]^+$	Na+ Adduct	Biological Source	Bioactivity	Reference
CZA-SHK-CC	Unidentified	$C_{16}H_{20}O_6Na$	3.813	331.115	-2.4	157.8971; 172.8592; 201.8864; 232.8278; 249.8306; 250.8333; 331.1142	N/A			
CZA-SHK-CC	Unidentified	$C_{16}H_{19}O_4$	6.127	275.1282	-0.4	81.0717; 117.0696; 145.0644; 151.0389; 153.0534; 173.059; 179.0339; 195.9122	N/A			
CZA-SHK-CC	Unidentified	$C_{28}H_{40}O_6$	7.4	473.2898	-1.1	305.7609; 349.7643; 387.2139; 388.2161	495.2724			
CZA-SHK-CC	Skeletocutin M	$C_{28}H_{42}O_6$	7.5	475.3067	1.5	261.7737; 313.1769; 349.7601; 371.2191; 439.2416	497.2876	*Skeletocutis* sp.	N/A	Cheng et al., 2019[14]
CZA-SHK-CC	Unidentified	$C_{24}H_{30}O_6$	7.7	415.2114	-1.7	303.1186; 321.7591	437.1937			
CZA-SHK-PLE	Unidentified	$C_{18}H_{28}O_{10}Na$	2.196	427.1579	-0.2	185.0880; 203.0508; 231.0453; 247.0927; 265.105; 293.7955; 307.1151	N/A			

Organism	Compound	Molecular Formula	Retention Time (Min)	*m/z* [M+H]⁺	ppm	MS/MS [M+H]⁺	Na+ Adduct	Biological Source	Bioactivity	Reference
CZA-SHK-PLE	Unidentified	$C_{38}H_{42}O_{17}$	2.6	771.2495	-0.6	112.0760; 155.0039; 181.0053; 183.0231; 226.0284; 238.0265; 293.0369; 370.0934	793.2316			
CZA-SHK-PLE	Unidentified	$C_{16}H_{26}O_7$	3.3	353.1575	-0.3	127.0353; 164.8744; 247.0933; 254.8670; 283.1148; 291.1555; 294.1376; 311.1417; 353.1573	N/A			
CZA-SHK-PLE	Unidentified	$C_{32}H_{30}O_4Na$	3.7	501.2064	4.4	168.0620; 210.0731; 240.0856; 254.101; 296.1100; 338.1213; 356.1321; 423.1705; 441.1799; 483.1985	N/A			
CZA-SHK-PYR	Unidentified	$C_{38}H_{42}O_{17}$	2.6	771.2497	-0.4	763.2244; 484.1093; 447.072; 402.0471; 345.0299; 276.0040; 222.0828	793.2322			

Organism	Compound	Molecular Formula	Retention Time (Min)	*m/z* [M+H]+	ppm	MS/MS [M+H]+	Na+ Adduct	Biological Source	Bioactivity	Reference
CZA-SHK-PYR	Unidentified	$C_{28}H_{40}O_6$	7.373	473.2888	-3.2		495.2724			
CZA-SHK-PYR	Clavilactone H	$C_{17}H_{18}O_4$	7.434	287.1282	-0.3	287.1277; 269.1182; 259.1328; 241.123; 219.0651	N/A	*Clitocybe clavipes*	Cytotoxicity	Sun et al., 2019[13]
CZA-SHK-PYR	Skeletocutin M	$C_{28}H_{42}O_6$	7.551	475.3086	5.5	439.2408; 371.2192; 349.756	497.2881	*Skeletocutis* sp.	N/A	Cheng et al., 2019[14]

References

1. Rukachaisirikul, V.; Rodglin, A.; Sukpondma, Y.; Phongpaichit, S.; Buatong, J.; Sakayaroj, J. Phthalide and isocoumarin derivatives produced by an *Acremonium* sp. isolated from a mangrove *Rhizophora apiculata*. *J. Nat. Prod.* **2012**, *75*, 853–858,doi:10.1021/NP200885E/SUPPL_FILE/NP200885E_SI_001.PDF.

2. Nakajima, H.; Kimura, Y.; Hamasaki, T. Spiciferinone, an azaphilone phytotoxin produced by the fungus *Cochliobolus spicifer*. *Phytochemistry* **1992**, *31*, 105–107, doi:10.1016/0031-9422(91)83016-E.

3. Kurobane, I.; Vining, L.C.; Gavin McInnes, A. Biosynthetic relationships among the secalonic acids isolation of emodin, endocrocin and secalonic acids from *Pyrenochaeta terrestris* and *Aspergillus aculeatus*. *J. Antibiot. (Tokyo).* **1979**, *32*, 1256–1266, doi:10.7164/antibiotics.32.1256.

4. Nguyen, D.M.T.; Do, L.M.T.; Nguyen, V.T.; Chavasiri, W.; Mortier, J.; Nguyen, P.P.K. Phenolic compounds from the lichen *Lobaria orientalis*. *J. Nat. Prod.* **2017**, *80*, 261–268, doi:10.1021/ACS.JNATPROD.6B00465/SUPPL_FILE/NP6B00465_SI_001.PDF.

5. El-Elimat, T.; Figueroa, M.; Raja, H.A.; Graf, T.N.; Swanson, S.M.; Falkinham, J.O.; Wani, M.C.; Pearce, C.J.; Oberlies, N.H. Biosynthetically distinct cytotoxic polyketides from *Setophoma terrestris*. *European J. Org. Chem.* **2015**, *2015*, 109–121, doi:10.1002/EJOC.201402984.

6. Oppong-Danquah, E.; Budnicka, P.; Blümel, M.; Tasdemir, D. Design of fungal co-cultivation based on comparative metabolomics and bioactivity for discovery of marine fungal agrochemicals. *Mar. Drugs* **2020**, *18*, doi:10.3390/md18020073.

7. Aly, A.H.; Debbab, A.; Clements, C.; Edrada-Ebel, R.; Orlikova, B.; Diederich, M.; Wray, V.; Lin, W.; Proksch, P. NF kappa B inhibitors and

antitrypanosomal metabolites from endophytic fungus *Penicillium* sp. isolated from *Limonium tubiflorum*. *Bioorg. Med. Chem.* **2011**, *19*, 414–421, doi:10.1016/J.BMC.2010.11.012.

8. Choi, J.H.; Ogawa, A.; Abe, N.; Masuda, K.; Koyama, T.; Yazawa, K.; Kawagishi, H. Chaxines B, C, D, and E from the edible mushroom *Agrocybe chaxingu*. *Tetrahedron* **2009**, *65*, 9850–9853, doi:10.1016/J.TET.2009.09.064.

9. Arnone, A.; Cardillo, R.; Nasini, G. Structures of Melleolides B-D, three antibacterial sesquiterpenoids from *Armillaria mellea*. *Phytochemistry* **1986**, *25*, 471–474, doi:10.1016/S0031-9422(00)85503-X.

10. Centko, R.M.; Williams, D.E.; Patrick, B.O.; Akhtar, Y.; Garcia Chavez, M.A.; Wang, Y.A.; Isman, M.B.; De Silva, E.D.; Andersen, R.J. Dhilirolides E-N, meroterpenoids produced in culture by the fungus *Penicillium purpurogenum* collected in Sri Lanka: structure elucidation, stable isotope feeding studies, and insecticidal activity. *J. Org. Chem.* **2014**, *79*, 3327–3335, doi:10.1021/JO4024039/SUPPL_FILE/JO4024039_SI_002.PDF.

11. Cai, S.; Sun, S.; Zhou, H.; Kong, X.; Zhu, T.; Li, D.; Gu, Q. Prenylated polyhydroxy-p-terphenyls from *Aspergillus taichungensis* ZHN-7-07. *J. Nat. Prod.* **2011**, *74*, 1106–1110, doi:10.1021/NP2000478/SUPPL_FILE/NP2000478_SI_001.PDF.

12. Wyatt, T.T.; Gerwig, G.J.; Kamerling, J.P.; Wösten, H.A.B.; Dijksterhuis, J. Structural analysis of novel trehalose-based oligosaccharides from extremely stress-tolerant ascospores of *Neosartorya fischeri* (*Aspergillus fischeri*). *Carbohydr. Res.* **2015**, *411*, 49–55, doi:10.1016/J.CARRES.2015.04.006.

13. Zhaocui, S.; Xudong, X.; Hanqiao, L.; Xinyi, X.; Guoxu, M.; Leiling, S. Five new meroterpenoids from the fruiting bodies of the Basidiomycete *Clitocybe clavipes* with cytotoxic activity. *Molecules* **2019**, *24*, 1–10, doi:10.3390/molecules24224015.

14. Cheng, T.; Chepkirui, C.; Decock, C.; Matasyoh, J.C.; Stadler, M. Skeletocutins M–Q: Biologically active compounds from the fruiting bodies of the Basidiomycete *Skeletocutis* sp. collected in Africa. *Beilstein J. Org. Chem.* **2019**, *15*, 2782–2789, doi:10.3762/bjoc.15.270.
